# High-dose polyethylene glycol-3350 and gatorade solutions for patients with previous inadequate bowel preparations for colonoscopy are safe and effective

**DOI:** 10.1186/s12876-023-02663-0

**Published:** 2023-05-11

**Authors:** David P. Gerard, Sophia F. Gerard, Manfred W. Raiser

**Affiliations:** Gastroenterology Services, Ltd., 3825 Highland Ave, Suite 203, Downers Grove, IL 60515 USA

**Keywords:** Endoscopy, PEG-3350, Gatorade, Colonoscopy preparation, Inadequate preparation

## Abstract

**Background:**

Inadequate bowel preparation for colonoscopy remains an issue resulting in lower adenoma detection rates and increased cost. We assessed the efficacy, safety and tolerability of high-dose bowel preparations in subjects who previously had an inadequate colonoscopy preparation.

**Methods:**

We performed a multi-step prospective trial of high-dose bowel preparations with subjects assigned to the dose higher than their previous inadequate preparation. Step 1: 1.5 times the standard-dose of polyethylene glycol 3350 (PEG, 459 g) and Gatorade; and Step 2: 2.0 times the standard-dose of PEG (612 g) and Gatorade, both were given as extended split-dose preparations. 69 outpatients consumed their preparation before a morning colonoscopy. The primary endpoint was colon cleanliness assessed by the Chicago bowel preparation scale (BPS). Safety was assessed by comparing a baseline basic metabolic panel (BMP) to a post-cleansing BMP. Patients with no history of inadequate colon cleansing who consumed standard doses of PEG (306 g to 357 g) and Gatorade were used as a comparison group. Tolerability of the bowel preparation was assessed using a subject-questionnaire.

**Results:**

When compared to controls consuming standard-dose bowel preparations, subjects consuming high-dose preparations had no statistically significant difference in colon cleanliness as measured by the modified or total Chicago BPS scores or differences in tolerability. Baseline and post-cleaning BMPs were not significantly different other than the BUN falling (*p* < 0.0001) after the preparation.

**Conclusions:**

The multi-step high-dose bowel cleansing protocol proved highly efficacious, safe and well tolerated in subjects who previously had an inadequate colonoscopy preparation.

*Trial registration*: ClinicalTrials.gov NCT02661750.

**Supplementary Information:**

The online version contains supplementary material available at 10.1186/s12876-023-02663-0.

## Background

An adequate bowel preparation for colonoscopy is necessary to visualize the mucosa and safely reach the cecum. Inadequate bowel cleansing remains an issue for up to 20% of those undergoing a colonoscopy [1] resulting in a lower adenoma detection rate and added cost [2]. Current guidelines [3] recommend that "consideration should be given to prescribing more aggressive preparations in patients who have a history of inadequate preparation quality" but no standardized recommendations exist for the subsequent bowel preparation.

A high percentage of those who had a previous inadequate preparation for colonoscopy have a subsequent inadequate preparation. A retrospective study [4] looked at 6990 patients of whom 307 (4.4%) had an inadequate preparation for colonoscopy. Of the 235 who had a subsequent colonoscopy, inadequate preparations occurred in 54 (23%) second colonoscopies, 10 of 38 (26%) third colonoscopies and 3 of 9 (33%) forth colonoscopies. This study was limited by the lack of data on how the initial inadequate preparations differed from those of the preparations used in the subsequent colonoscopies.

Two studies [5, 6] have examined "intensive" bowel preparations in those with previous inadequate preparations, but the "intensive" bowel preparations were no different than standard-dose regimens in use today and 10–33% of subjects had inadequate preparations with many of the "adequately" cleansed colons containing residual fecal material. No studies have investigated high-dose bowel preparations in this population.

In the United States, pure PEG-3350 powder is sold over-the-counter by the gram in sachets (packages) of 17 g or bottles ranging in size from 119 to 765 g for use as a laxative with a recommended dose of 17 g. PEG-3350 with electrolytes is sold by prescription for cleansing the colon for colonoscopy and is rarely used for constipation. In other parts of the world, PEG-4000 is sold as a laxative but there has never been a comparison of the osmotic effect of PEG-3350 vs. PEG-4000 when used a laxative in humans [7].

A number of studies [8] have reported using lower doses (238 g to 255 g) of pure PEG-3350 (PEG) and Gatorade (The Gatorade Company, Chicago, IL) given as a split-dose or the day-prior and found inadequately cleaned colons in 2% to 18% of subjects. We previously showed [8] that 306 g of PEG and Gatorade given as a split-dose and 357 g of PEG and Gatorade given the day-prior (68 g PEG at noon and 289 g the evening-prior) were equally efficacious with only 1.5% of colons inadequately cleansed (defined as less than 95% of the mucosa seen) and well tolerated by subjects who were able to consume 98.6% of their preparation solutions.

Since 2008, our clinic almost exclusively used moderate (306 g to 357 g) doses of PEG in 1.9 l (64 oz) of Gatorade as our standard colonoscopy preparations. For those whose previous preparations were almost adequate, we use 374 g of PEG and Gatorade given as a split-dose. For those whose previous preparations were fair or poor, we use high doses (459 g to 612 g) of PEG and Gatorade with a dose chosen that was higher than the previous failed dose and given as a split dose over an extended period of time. This extended high-dose cleansing protocol seemed to have a high efficacy and be well tolerated.

This prospective study reports the efficacy of a multi-step escalating high-dose extended cleansing protocol for those whose previous colon preparations were inadequate. The primary objectives of this study were to examine the efficacy and safety of high-dose PEG and Gatorade preparations for a morning colonoscopy. Secondary objectives of this study were to examine tolerability and patient acceptance of high-dose bowel preparations.

## Methods

### Study design and oversight

The protocol was approved by the AMITA Institutional Review Board (AMITA 2015-0004-02, ClinicalTrials.gov NCT02661750 first registered January 22, 2016). The trial was designed by the authors with no outside input. All authors contributed to the study conception and design, material preparation, data collection and analysis. The first draft of the manuscript was written by David P. Gerard and all authors commented on each version of the manuscript. All authors read and approved the final manuscript. AMITA waived the fee for the Institutional Review Board. All other expenses associated with this study were paid for by the authors. Subjects received no financial reward and paid for their own preparations.

The study was prospective and non-blinded. All patients in our clinic who previously had an inadequate colonoscopy preparation with less than 90% of the mucosa seen and scheduled a subsequent colonoscopy between January 2016 and December 2020 where invited to participate. All colonoscopies were performed by one of 2 gastroenterologists (DPG, MWR) between 8 a.m. to noon in the endoscopy labs at our office or two community hospitals.

Subjects were outpatients undergoing elective colonoscopy, at least 18 years of age and not pregnant. Each subject signed an informed consent document.

Subjects were assigned to receive a high-dose (459 g or 612 g) PEG and Gatorade bowel preparation solution dosed as shown in the upper half of Table 1 (the standard of care for our practice) with the ingredients of those solutions shown in the lower half of Table 1. The dose of the solution chosen was one step higher than the step that was closest to the total dose of the bowel preparation solution consumed previously. For example, if a subject had previously failed a preparation of 374 g of PEG and Gatorade which is 1.22 times our standard dose of 306 g of PEG, they would have been assigned to a Step 1 (1.5 times the standard-dose of PEG, 459 g) preparation. If a subject had failed 5.5 L of PEG-ELS which is 1.37 times our standard dose of 4 l, they would have been assigned to a Step 2 (2.0 times the standard-dose of PEG, 612 g) preparation.

**Table 1:** Three-step high-dose bowel preparation protocol (top) with the contents of the bowel preparations obtained from the package inserts (below) compared to 4 L SF PEG-ELS (NuLYTELY) and our standard preparation of 306 g PEG in 64 oz of Gatorade

	Bowel preparation solution				
	4 L SF-PEG-ELS (TriLyte or NuLYTELY)	64 oz Gatorade + 306 g PEG Standard Dose	96 oz Gatorade + 459 g PEG Step 1	128 oz Gatorade + 612 g PEG Step 2	160 oz Gatorade + 765 g PEG Step 3
Dose					
Multiple of standard dose		1.0	1.5	2.0	2.5
Noon one day-prior to colonoscopy			51 g PEG + 12 oz Clear Liquid	153 g PEG + 32 oz Gatorade	306 g PEG + 32 oz Gatorade
6 PM one day-prior to colonoscopy	2 L	153 g PEG + 32 oz Gatorade	255 g PEG + 32 oz Gatorade	306 g PEG + 32 oz Gatorade	306 g PEG + 32 oz Gatorade
4 h prior to colonoscopy	2 L	153 g PEG + 32 oz Gatorade	153 g PEG + 32 oz Gatorade	153 g PEG + 32 oz Gatorade	153 g PEG + 32 oz Gatorade
Contents					
Sodium (mmol)	260	38	57	76	95
Potassium (mmol)	20	6	9	12	15
Chloride (mmol)	212	20	30	40	50
Bicarbonate (mmol)	68				
Phosphate (mmol)		6	9	12	15
Citrate (mmol)		More than 6	More than 9	More than 12	More than 15
PEG (mmol)	125	91	136	182	227
PEG (g)	420	306	459	612	765
Fructose/Sucrose (g)		112	168	224	280
Water (ml)	4000	1893	3370	4494	5617
Poorly Absorbable mOsm	275	206	309	412	515
Cost ($)	20.92	14.92	15.92	29.84	30.84

Poorly Absorbed mOsm is estimated by adding the number of mOsms of Phosphate plus 2.2 multiplied by the mOsm of PEG [12]. The actual number mOsm reaching the colon may differ from this estimate since some phosphate is absorbed and the effective osmolarity of PEG rises more than expected as its concentration increases. Water includes the volume of the preparation and any clear liquids required to be consumed with the preparation. Cost data for patients paying cash at an Illinois Wal-Mart in January 2021 for the least expensive generic version

L indicates liters; mOsm, milliosmoles. SF, sulfate free; ELS, electrolyte solution; PEG, polyethylene glycol-3350

All patients were instructed to omit fruits and vegetables with seeds or foods that are high in fiber from their diet for 3 days-prior to their colonoscopy and to consume a clear liquid diet starting the day-prior (our standard dietary instructions for all patients undergoing colonoscopy). The patient instructions for the bowel preparations are provided in Additional file 1. A gastroenterologist provided face-to-face education on the bowel preparation protocol and the physician on-call was available to answer any questions during the preparation for all control subjects, the 59 subjects who had their previous inadequate preparation in our clinic and all subjects consuming a study preparation. It is unknown what education was received by the 10 subjects who had their previous inadequate preparation elsewhere.

The study was designed to include a total of up to 100 subjects, but recruitment would terminate after 5 years if at least 50 subjects had enrolled. A subject was allowed to withdraw from the study until the day-prior to their colonoscopy. Beginning the day-prior to the colonoscopy, all subjects were evaluated on an intention-to-treat basis no matter what bowel preparation they actually used. Subjects could be switched to another bowel cleaning regimen chosen by the physician on-call if they could not tolerate the one assigned to them.

A physician (DPG) was responsible for monitoring the data for any adverse outcomes.

### Measurements

A questionnaire was filled out by each subject immediately before the colonoscopy is provided in Additional file 2.

A questionnaire was filled out by the gastroenterologist immediately after the colonoscopy rating the cleanliness of the bowel preparation and is provided in Additional file 3.

A primary endpoint of this study (Table 2) was the cleanliness of the colon on the Chicago bowel preparation scale [9] (Chicago BPS). Another primary end point was the safety of the study preparations as assessed by comparing the sodium, potassium, chloride, carbon dioxide, BUN, creatinine, glucose and calcium from a baseline basic metabolic profile (BMP) obtained within 3 months prior to the colonoscopy and a BMP obtained the morning of the colonoscopy, after the preparation had been consumed and just before the IV was started.

**Table 2:** The Chicago Bowel Preparation Scale [8] and the Boston Bowel Preparation Scale [9]

Chicago Bowel Preparation Scale [8]	
Rating for each colon segment	
0 = Unprepared colon segment with stool that cannot be cleared (> 15% of the mucosa not seen)	
5 = Portion of mucosa in segment seen after cleaning, but up to 15% of the mucosa not seen due to retained material	
10 = Minor residual material after cleaning, but mucosa of segment generally well seen	
11 = Entire mucosa of segment well seen after cleaning	
12 = Entire mucosa of segment well seen without washing (suctioning of liquid allowed)	
Rating For the Amount of Fluid in the Whole Colon	
3 = Large amount of fluid (> 300 cc)	
2 = Moderate amount of fluid (151–300 cc)	
1 = Minimal amount of fluid (51–150 cc)	
0 = Little fluid (≤ 50 cc)	
Chicago BPS Total Score	
Calculated by adding the scores of the right, transverse and left colon segments. The Chicago BPS Total Score ranges from 0 (very poor) to 36 (outstanding) with adequate preparations have scores greater than 24. The score for the fluid in the whole colon is reported separately	
Modified Chicago BPS Score	
Calculated by adding the scores of the right, transverse and left colon segments after collapsing the best segment of 12 and 11 into a score of 11. The Modified Chicago BPS score ranges from 0 (very poor) to 33 (outstanding). The score for the fluid in the whole colon is reported separately	
Boston Bowel Preparation Scale [9]	
Rating for each colon segment	
0 = Unprepared colon segment with stool that cannot be cleared	
1 = Portion of mucosa in segment seen after cleaning, but other areas not seen due to retained material	
2 = Minor residual material after cleaning, but mucosa of segment generally well seen	
3 = Entire mucosa of segment well seen after cleaning	
Boston BPS Total Score	
Calculated by adding the scores of the right, transverse and left colon segments. The Boston BPS total score ranges from 0 (very poor) to 9 (excellent). Adequate preparations have three segment scores of 2 or 3	

Secondary end points of the study were the tolerance of high-dose bowel preparations determined from the subject questionnaire, cleanliness of the colon on the Boston Preparation Scale [10] (Boston BPS) and 3 measures of cleanliness reported on an adequate/inadequate scale: (1) a dichotomous adequate/inadequate scale [9] (adequate/inadequate BPS) in which adequate was defined as being able to see at least 95% of the colon mucosa after cleansing maneuvers; (2) a Chicago BPS total score [9] of less than 25; and (3) any Boston BPS segments score [10] of 0 or 1.

400 subjects from arm 2 (357 g of PEG and Gatorade given the day-prior) and arm 3 (306 g of PEG and Gatorade given as a split-dose) of a previous colon cleansing study [8] who consumed standard doses of PEG and Gatorade and had no history of inadequate preparations were used as a comparison group. The subject and gastroenterologist questionnaires from this prior study were almost identical to the ones used in this study.

Before the study began, each participating gastroenterologist watched a video (domweb.bumc.bu.edu/bowelprep/instruction.php) as a calibration exercise for both the Chicago BPS and Boston BPS with the results discussed among the authors.

### Data analysis

Continuous variables were compared using Student’s t-test (unpaired, two-tailed). Categoric variables with 2 outcomes were compared using the Fisher's exact test (two-tailed). Categoric variables with more than 2 outcomes were compared using Pearson’s Chi-squared test (non-directional).

### Power calculation

A power calculation was performed as part of the study design process**.** With a total of 50 patients entered into the study, the probability is 80 percent that the study will detect a difference at a two-sided 0.05 significance level: (1) if the true difference between serum sodium measurements before and after the preparation is 2.0 mmol/l (based on the assumption that the standard deviation of the serum sodium measurements is 2.5); and (2) if the true difference between serum potassium measurements is 0.28 meq/dl (based on the assumption that the standard deviation of the serum potassium measurements is 0.43). (3) if the true difference between Total Chicago BPS scores of the control patients and study patients is 1.66 (based on the assumption that the standard deviation of the Total Chicago BPS scores is 3.94).

## Results

### Subjects and endoscopic findings

During recruitment period from January 2015 to December 2020, 79 outpatient colonoscopies were scheduled in our clinic for patients who previously had inadequate preparations and all these patients were eligible to participate in the study. One patient declined to participate leaving 78 who enrolled. 9 patients withdrew at least 24 h before their scheduled colonoscopy including 2 whose insurance changed, 2 who passed away from non-gastrointestinal causes, 1 whose health declined and chose not undergo a colonoscopy, 1 who had his colonoscopy with another doctor and 3 who withdrew for unstated reasons. This left 69 subjects who consumed a study preparation and were analyzed for this study. One Step 2 (2.0 times the standard-dose of PEG) subject had only one previous inadequate preparation because he mistakenly consumed 510 g of PEG (rather than 306 g) with his first preparation.

The baseline characteristics are shown in Table 3 for all 69 subjects, for the 59 subjects who used Step 1 (1.5 times the standard-dose of PEG) and 10 subjects who used Step 2 (2.0 times the standard-dose of PEG) preparations along with similar data from 400 historical control subjects. No subjects required a Step 3 preparation (2.5 times the standard-dose of PEG). Compared to historical controls, subjects were significantly older (65.3 ± 9.4 vs. 60.2 ± 11.9 years old, *p* = 0.0008), were less likely to be undergoing a screening examination (17.4% vs. 43.2%, *p* < 0.0001) and more likely to have polyps found (53.6% vs. 40.5%, *p* = 0.048).

**Table 3:** Data from the questionnaire filled out by the gastroenterologists describing the baseline characteristics of the subjects, the findings and all the previous failed colonoscopy preparations

	Study preparations			Control	*P* value statistical significance	
	All	Step 1	Step 2	306 g PEG + G		
	Step 1 + 2	459 g PEG + G	612 g PEG + G	357 g PEG + G	All ↔ CONTROL	Step 1 ↔ Step 2
N	69	59	10	400		
Sex ratio (M:F)	38:31:00	31:28:00	07:03	200:200	NS	NS
MD (DPG:MWR:JLH)	52:17:00	44:15:00	08:02:00	201:98:101	NS	NS
Age in years (mean ± SD)	65.3 ± 9.4	65.5 ± 9.4	64.7 ± 10.0	60.2 ± 11.9	*p* = 0.0008	NS
Race						
Caucasian	61 (88.4%)	51 (86.4%)	10 (100.0%)	340 (85.5%)	NS	NS
African American	3 (4.3%)	3 (5.1%)	0	18 (5.5%)	NS	NS
Hispanic	2 (2.9%)	2 (3.4%)	0	17 (2.0%)	NS	NS
Other	3 (4.3%)	3 (5.1%)	0	25 (7.0%)	NS	NS
Procedure						
Performed in Our Office	52 (75.4%)	43 (72.9%)	9 (90.0%)	268 (60.5%)	NS	NS
Start Time (mean hour ± SD)	10.3 ± 0.9	10.3 ± 0.9	10.2 ± 1.0	10.3 ± 0.9	NS	NS
Previous Colon Resection	4 (5.8%)	2 (3.4%)	2 (20.0%)	5 (1.2%)	NS	NS
Indications (multiple indications possible)						
Screening	12 (17.4%)	11 (18.6%)	1 (10.0%)	173 (43.2%)	*p* < 0.0001	NS
History of Polyps	45 (65.2%)	39 (66.1%)	6 (60.0%)	95 (23.7%)	*p* < 0.0001	NS
History of Colon Cancer	3 (4.3%)	2 (3.4%)	1 (10.0%)	6 (1.5%)	NS	NS
FH Colon Neoplasia	14 (20.3%)	12 (20.3%)	2 (20.0%)	65 (16.2%)	NS	NS
Rectal Bleeding	3 (4.3%)	3 (5.1%)	0	63 (15.7%)	*p* = 0.008	NS
Anemia	2 (2.9%)	2 (3.4%)	0	7 (1.7%)	NS	NS
Other	3 (4.3%)	1 (1.7%)	0	27 (6.7%)	NS	NS
Findings (multiple findings possible)						
Normal	10 (14.5%)	9 (15.2%)	1 (10.0%)	168 (42.0%)	*p* < 0.0001	NS
Colon Polyps	37 (53.6%)	31 (52.5%)	6 (60.0%)	162 (40.5%)	*p* = 0.048	NS
Colon cancer	2 (2.9%)	2 (3.4%)	0	4 (1.0%)	NS	NS
Diverticulosis	36 (52.2%)	30 (50.8%)	6 (60.0%)	114 (28.5%)	*p* = 0.0002	NS
Crohn’s disease	0	0	0	6 (1.5%)	NS	NS
Ulcerative colitis	2 (2.9%)	1 (1.7%)	1 (10.0%)	5 (1.2%)	NS	NS
Other colitis	2 (2.9%)	2 (3.4%)	1 (10.0%)	3 (0.7%)	NS	NS
Incomplete colonoscopy	3 (4.3%)	3 (5.1%)	0	1 (0.2%)	*p* = 0.01	NS
Previous colonoscopy performed in our clinic	59 (85.5%)	49 (83.0%)	10 (100.0%)			NS
Previous inadequate preparations (mean ± SD)	1.55 ± 0.81	1.46 ± 0.82	2.10 ± 0.57			*p* = 0.02
1 Inadequate Preparation	43 (72.9%)	42 (85.7%)	1 (10.0%)			*p* = 0.0004
2 Inadequate Preparations	16 (27.1%)	9 (18.4%)	7 (70.0%)			*p* = 0.0008
3 Inadequate Preparations	8 (13.6%)	5 (10.2%)	2 (20.0%)			NS
4 Inadequate Preparations	2 (2.9%)	2 (3.4%)	0			NS
Previous failed preparations (dosing)						
PEG-ELS 4 L						
Day-Prior	22	19	3			NS
Unknown	17	14	3			NS
PEG + Gatorade						
Day-Prior 306 g PEG	24	21	3			NS
Day-Prior 357 g PEG	17	14	3			NS
Split 306 g PEG	8	7	1			NS
Unknown	2	2	0			NS
Split 459–510 g PEG	8	0	8			*p* < 0.0001
PEG-Asc 2 L						
Split	1	1	0			NS
NaPhospate Tablets						
Split (dose unknown)	1	1	0			NS
Unknown						
Unknown	7	6	1			NS
Last failed preparation description						
Poor or Less than 80% of Mucosa Seen	27	24	3			NS
80–89% of the Mucosa Seen	18	15	3			NS
Fair or Inadequate or Suboptimal	24	20	4			NS

*P* value statistical significance shows arms that were statistically different with *p* < 0.05. The controls were from a previous colon cleansing study [8] that looked at 306 g of PEG given as a split-dose and 357 g of PEG given the day-prior both with Gatorade in subjects with no history of inadequate colon preparations

NS indicates no significant differences; PEG + G, polyethylene glycol 3350 and Gatorade; ELS, electrolyte solution; Asc, ascorbic acid

Data from the questionnaire filled out by the gastroenterologists describing the previous failed colonoscopy preparations are shown in Table 3 for all subjects and for subjects who used Step 1 and Step 2 preparations. Subjects who previously had 1, 2, 3 or 4 inadequate preparations were 43 (72.9%), 16 (27.1%), 8 (13.6%) and 2 (3.4%) respectively**.**

There were no major adverse events noted immediately after the colonoscopies or reported by subjects after discharge from the endoscopy labs.

### Preparation quality

Colon cleanliness data is shown in Table 4 for all subjects, for subjects who used Step 1 and Step 2 preparations along with similar data from 400 control subjects. One Step 1 subject did not have their right and transverse colon segments rated due to an incomplete colonoscopy. When compared to controls, subjects had no statically significant differences in the Chicago BPS, Modified Chicago BPS, Boston BPS or Adequate/Inadequate BPS scores other than subjects having more perfect scores of 36 on Chicago BPS (82.3% vs 55.4%, *p* < 0.0001) and higher Fluid Total Colon scores signifying wetter colons (2.25 ± 0.77 vs 1.43 ± 0.98, *p* < 0.0001).

**Table 4:** Colon cleanliness data from the questionnaire filled out by the gastroenterologists by preparation arm

	Study preparations			Control	*P* value statistical significance	
	All	Step 1	Step 2	306 g PEG + G		
	Step 1 + 2	459 g PEG + G	612 g PEG + G	357 g PEG + G	All ↔ CONTROL	Step 1 ↔ Step 2
*Chicago BPS*						
Right Colon (mean ± SD)	11.49 ± 1.87	11.43 ± 2.02	11.80 ± 0.42	11.33 ± 1.46	NS	NS
TV Colon (mean ± SD)	11.51 ± 1.88	11.45 ± 2.03	11.90 ± 0.32	11.55 ± 1.24	NS	NS
Left Colon (mean ± SD)	11.49 ± 1.51	11.42 ± 1.62	11.90 ± 0.32	11.45 ± 1.54	NS	NS
Total (mean ± SD)	34.49 ± 4.86	34.30 ± 6.08	34.60 ± 0.97	34.33 ± 3.94	NS	NS
Total Exactly 36	56 (82.3%)	48 (82.8%)	8 (80.0%)	221 (55.4%)	*p* < 0.0001	NS
Fluid Total Colon (mean ± SD)	2.25 ± 0.77	2.31 ± 0.75	1.9 ± 0.88	1.43 ± 0.98	*p* < 0.0001	NS
*Chicago BPS (modified)*						
Right Colon (mean ± SD)	10.65 ± 1.66	10.58 ± 1.81	11.00 ± 0.00	10.72 ± 1.25	NS	NS
TV Colon (mean ± SD)	10.63 ± 1.67	10.56 ± 1.81	11.00 ± 0.00	10.79 ± 1.02	NS	NS
Left Colon (mean ± SD)	10.65 ± 1.25	10.59 ± 1.35	11.00 ± 0.00	10.72 ± 1.32	NS	NS
Total (mean ± SD)	31.93 ± 4.16	31.73 ± 4.53	33.00 ± 0.00	32.23 ± 3.33	NS	NS
Total Exactly 33	61 (89.7%)	51 (87.9%)	10 (100.0%)	342 (85.7%)	NS	NS
*Boston BPS*						
Right Colon (mean ± SD)	2.90 ± 0.43	2.88 ± 0.46	3.00 ± 0.00	2.86 ± 0.43	NS	NS
TV Colon (mean ± SD)	2.87 ± 0.45	2.84 ± 0.49	3.00 ± 0.00	2.89 ± 0.38	NS	NS
Left Colon (mean ± SD)	2.81 ± 0.52	2.78 ± 0.56	3.00 ± 0.00	2.87 ± 0.44	NS	NS
Total (mean ± SD)	8.58 ± 1.30	8.50 ± 1.39	9.00 ± 0.00	8.61 ± 1.16	NS	NS
Total Exactly 9	61 (89.7%)	51 (87.9%)	10 (100.0%)	344 (86.2%)	NS	NS
*Inadequate preparation*						
Adequate/inadequate BPS	3 (4.3%)	3 (5.1%)	0	6 (1.5%)	NS	NS
Chicago BPS total < 25	3 (4.3%)	3 (5.1%)	0	6 (1.5%)	NS	NS
Boston BPS Any Segment 0 or 1	3 (4.3%)	3 (5.1%)	0	6 (1.5%)	NS	NS
Total amount of fluid aspirated (ml ± SD)	406 ± 282	404 ± 272	419 ± 354			NS
Total amount of washing fluid (ml ± SD)	62 ± 195	59 ± 197	84 ± 193			NS

*P* value statistical significance shows arms that were statistically different with *p* < 0.05. The controls were from a previous colon cleansing study [8] that looked at 306 g of PEG given as a split-dose and 357 g of PEG given the day-prior both with Gatorade in subjects with no history of inadequate colon preparations

NS indicates no significant differences; PEG + G, polyethylene glycole-3350 and Gatorade

### Preparation tolerance and safety by subjects

Data from the questionnaire filled out by the subjects is shown in Table 5 for all subjects, for subjects who used Step 1 and Step 2 preparations along with similar data from 400 control subjects. When compared to controls, subjects had no statically significant differences in measures of tolerance including the difficulty of drinking the preparation but subjects were less likely to rate the overall difficulty of the preparation as easy (52.2% vs. 63.0%, *p* = 0.02).

**Table 5:** Data from the questionnaire filled out by the subjects by preparation arm

	Study preparations			Control	*P* value statistical significance	
	All	Step 1	Step 2	306 g PEG + G		
	Step 1 + 2	459 g PEG + G	612 g PEG + G	357 g PEG + G	All ↔ CONTROL	Step 1 ↔ Step 2
*Side effects*						
Nausea (N:Mild:Mod:S)	44:18:5:2	38:15:4:2	6:3:1:0	254:105:28:13	NS	NS
Vomiting (N:Mild:Mod:S)	68:0:1:0	58:0:1:0	10:0:0:0	365:15:11:9	NS	NS
Ab. Cramps/Pain (N:Mild:Mod:S)	44:21:3:1	39:17:2:1	5:4:1:0	262:123:14:4	NS	NS
Bloating (N:Mild:Mod:S)	24:33:11:1	19:29:10:1	5:4:1:0	152:189:54:5	NS	NS
*Other side effects*						
Headache	3 (4.3%)	3 (5.0%)	0	12 (3.0%)	NS	NS
Faint/Lightheaded	1 (1.4%)	1 (1.0%)	0	3 (0.7%)	NS	NS
Chills	1 (1.4%)	1 (2.0%)	0	6 (1.5%)	NS	NS
Heartburn	1 (1.4%)	1 (2.0%)	0	5 (1.2%)	NS	NS
Other	0	0	0	2 (0.5%)	NS	NS
% Preparation Consumed (mean ± SD)	98.9 ± 5.1	98.7 ± 5.5	100.0 ± 0.0	98.6 ± 5.4	NS	NS
Consumed 100% of Preparation	63 (91.3%)	53 (89.8%)	10 (100.0%)	367 (91.7%)	NS	NS
Incomplete % Consumed (mean ± SD)	87.2 ± 13.0	87.2 ± 13.0		84.4 ± 10.4	NS	
Called MD for Advice	5 (7.2%)	4 (6.8%)	1 (10.0%)	18 (4.5%)	NS	NS
Needed Fleets Enemas	9 (13.0%)	6 (10.2%)	3 (30.0%)	42 (10.5%)	NS	NS
Number of Enemas Needed (mean ± SD)	1.67 ± 0.50	1.67 ± 0.52	1.67 ± 0.58	1.79 ± 0.87	NS	NS
Changed Preparation Due to Intolerance	0	0	0	2 (0.5%)	NS	NS
Woke Up at Night for Bowel Movement	50 (72.5%)	42 (71.2%)	8 (80.0%)	245 (61.2%)	NS	NS
For a Future Colonoscopy Use the Same Preparation Again (Yes)	59 (85.5%)	50 (84.7%)	9 (90.0%)	352 (88.0%)	NS	NS
*How difficult was*						
It To Drink the Liquid (E:Mild:Mod:VD)	36:17:13:3	30:16:10:3	6:1:3:0	242:112:40:6	NS	NS
The Overall Preparation (E:Mild:Mod:VD)	36:18:12:3	30:17:9:3	6:1:3:0	252:109:35:4	*p* = 0.02	NS
Had Previous Colonoscopy	69 (100.0%)	50 (100.0%)	10 (100.0%)	258 (64.5%)	*p* < 0.0001	NS
How Compare to Previous Preparation (E:S:M)	28:33:06	25:28:04	03:05:02	170:64:24	*p* = 0.0004	NS
Take Medication for Constipation	18 (26.1%)	15 (25.4%)	3 (30.0%)			NS

*P* value statistical significance shows arms that were statistically different with *p* < 0.05. The controls were from a previous colon cleansing study [8] that looked at 306 g of PEG given as a split-dose and 357 g of PEG given the day-prior both with Gatorade in subjects with no history of inadequate colon preparations

For “Side Effects,” N = None, Mild = Mild, Mod = Moderate or S = Severe. For “How Difficult was,” E = Easy, Mild = Mildly Difficult, Mod = Moderately Difficult or VD = Very Difficult. For “Previous Preparation,” the study preparation was E = Easier, S = Same or M = More Difficult compared to the previous preparation. NS indicates no significant differences; PEG + G, polyethylene glycole-3350 and Gatorade

Baseline and post-preparation BMPs are shown in Table 6 for all subjects and for subjects who used Step 1 and Step 2 preparations. Hemolyzed samples and other lab errors (all in Step 1 subjects with normal renal function) resulted in 66 to 68 pairs of baseline and post-preparation values from the BMPs available for analysis. When the baseline BMPs were compared to the BMPs drawn as the IV was started and after subjects had consumed their preparation, there were no significant difference in any of the electrolytes, glucose or creatinine measurements but the BUN was significantly lower after the preparation (16.7 ± 9.6 vs. 11.7 ± 6.8, *p* = 0.0006).

**Table 6:** Data from basic metabolic profile obtained at baseline (Pre) and on the morning of the colonoscopy (Post) just as the IV was being started

	Study preparations						*P* value statistical significance		
	All (Step 1 + Step 2)		Step 1 (459 g PEG + G)		Step 2 (612 g PEG + G)		All	Step 1	Step 2
	Pre	Post	Pre	Post	Pre	Post	Pre ↔ Post	Pre ↔ Post	Pre ↔ Post
All Subjects (n = 69)									
Na (mmol/l) (mean ± SD)	140.0 ± 2.7	139.7 ± 3.1	140.2 ± 2.4	139.8 ± 2.9	138.3 ± 3.9	139.2 ± 4.2	NS	NS	NS
K (mmol/l) (mean ± SD)	4.36 ± 0.41	4.24 ± 0.47	4.35 ± 0.43	4.21 ± 0.49	4.43 ± 0.28	4.39 ± 0.32	NS	NS	NS
Cl (mmol/l) (mean ± SD))	103.5 ± 4.1	103.5 ± 3.7	103.6 ± 3.7	103.6 ± 3.3	102.7 ± 6.1	102.7 ± 5.4	NS	NS	NS
CO2 (mmol/l) (mean ± SD)	25.6 ± 2.6	25.1 ± 3.0	25.6 ± 2.6	25.4 ± 3.0	25.7 ± 2.7	23.5 ± 2.8	NS	NS	NS
BUN (mg/dl) (mean ± SD)	16.7 ± 9.6	11.7 ± 6.8	16.6 ± 10.0	11.7 ± 7.1	17.4 ± 7.1	12.0 ± 5.6	*p* = 0.0006	*p* = 0.003	NS
Cre (mg/dl) (mean ± SD)	0.96 ± 0.45	0.92 ± 0.40	0.96 ± 0.48	0.91 ± 0.42	0.97 ± 0.26	0.95 ± 0.25	NS	NS	NS
Glucose (mg/dl) (mean ± SD)	115 ± 49	119 ± 50	117 ± 52	120 ± 47	107 ± 23	118 ± 71	NS	NS	NS
Ca (mg/dl) (mean ± SD)	9.26 ± 0.49	9.10 ± 0.46	9.23 ± 0.51	9.10 ± 0.47	9.42 ± 0.34	9.14 ± 0.41	NS	NS	NS
Na < 130 mmol/l	0	0	0	0	0	0			
K < 3.3 mmol/l	0	0	0	0	0	0			
Subjects with baseline Cre > 1.29 (n = 7)									
BUN (mmol ± SD)	36.0 ± 18.1	26.4 ± 10.9	37.0 ± 19.6	27.3 ± 11.7	30	21	NS	NS	
Creatinine (mmol ± SD)	1.96 ± 0.83	1.81 ± 0.67	2.05 ± 0.87	1.89 ± 0.70	1.41	1.34	NS	NS	

7 Individual Subjects with baseline Cre > 1.29									
		Subject 1	Subject 2	Subject 3	Subject 4	Subject 5	Subject 6	Subject 7	
		Pre ↔ Post	Pre ↔ Post	Pre ↔ Post	Pre ↔ Post	Pre ↔ Post	Pre ↔ Post	Pre ↔ Post	
BUN (mmol ± SD)		22 ↔ 21	30 ↔ 21	27 ↔ 19	35 ↔ 32	33 ↔ 24	29 ↔ 19	76 ↔ 49	
Creatinine (mmol ± SD)		1.30 ↔ 1.24	1.41 ↔ 1.34	1.49 ↔ 1.39	1.67 ↔ 1.50	1.73 ↔ 1.67	2.48 ↔ 2.81	3.62 ↔ 2.73	

*P* value statistical significance shows arms that were statistically different with *p* < 0.05

NS indicates no significant differences

## Discussion

Our prospective, non-blinded study of high doses of PEG (459 g to 612 g) and Gatorade given as part of a multi-step escalating extended cleansing protocol is the first to report the efficacy, tolerance and safety of any high-dose preparation for subjects whose colons were inadequately cleansed for a previous colonoscopy.

Compared to historical control subjects who had no history of inadequate preparations who consumed standard doses of PEG (306 g to 357 g) and Gatorade, subjects had no significant difference in the colon cleanliness as measured by any of the bowel preparation scales including: (1) the Chicago BPS total score [11] which is a measure of mucosal visualization and effort required to wash the colon and a primary end point of this study; (2) the modified Chicago BPS scores and Boston BPS [12] scores which are measures of mucosal visualization after cleansing maneuvers; and (3) all 3 measures of adequate/inadequate cleansing. Subjects had significantly higher Chicago BPS fluid scores indicating their colons required more liquid to be aspirated compared to controls.

Our definition of adequate cleansing (seeing 95% of the colonic mucosa after cleaning maneuvers) is better defined and sets a higher threshold than many other bowel preparation studies making our findings even more clinically relevant. It is important to note that the quality of the preparations was not just adequate but outstanding. All 10 subjects who used a Step 2 preparation (2.0 times the standard-dose of PEG) had perfect scores on the modified Chicago BPS and Boston BPS. Of the 59 subjects who used a Step 1 preparation (1.5 times the standard-dose of PEG): (1) 51 (86.4%) were not felt to require a higher dose of their bowel cleansing solutions for their next colonoscopy; and (2) 8 were felt to require a Step 2 preparation for their next colonoscopy including 5 (8.5%) who required extensive cleaning maneuvers to make their preparations adequate and 3 (4.3%) had inadequate preparations despite the cleaning maneuvers. Compared to historical controls, subjects had significantly more perfect Chicago BPS total scores of 36 (colons that needed no washing to visualize the mucosa).

Another primary end point of this study was the safety of high doses of bowel cleansing preparations. The doses of PEG used as part of our Step 1 (459 g) and Step 2 (612 g) preparations are 1.5 to 2 times higher than our standard moderate split-dose of (306 g) PEG and Gatorade and 1.9 to 2.6 times higher than low doses (238 g to 255 g) of PEG and Gatorade. Unlike PEG-ELS, Gatorade is a hypotonic electrolyte solution (Table 1) and consuming 1.5 to 2.0 times the standard-dose of Gatorade might cause hyponatremia or hypokalemia.

When the baseline BMPs were compared to the BMPs drawn as the IV was started and after subjects had consumed their preparation (Table 6), there were no significant difference in any of the electrolytes, glucose or creatinine measurements but the BUN was significantly lower after the preparation. No subjects developed hyponatremia or hypokalemia. Subgroup analyses of the 7 subjects with elevated baseline creatinine between 1.30 mg/dl and 3.62 mg/dl found no significant difference in any of electrolytes, BUN or creatinine after the preparation with all having a lower BUN and 6 of 7 having a lower creatinine after the preparation.

Secondary endpoints of this study were tolerability and patient acceptance of high-dose bowel preparations. Including historical controls allowed us to compare the tolerance of high-dose preparations compared to standard dose preparations. The study preparations were well tolerated by all measures with subjects able to consume 98.9% of their high-dose cleansing solutions which was not significantly different than controls who consumed standard doses. Compared to controls, subjects had no significant increase in side effects, were just as willing to use the same preparation again for future colonoscopies, did not find the liquid more difficult to drink than their previous preparation, but did find the overall preparation more difficult.

In order to understand the clinical relevance of studies such as this one that look at difficult to cleanse colons, it is necessary to understand the efficacy of the failed preparations and how selective the criteria were for inclusion. If the failed preparations were only 80% efficacious, then switching those with inadequate preparations to a standard preparation that is 98% efficacious would be expected to cleanse 90% of colons adequately (assuming the same patients would fail both preparations). Studies enrolling patients with a single prior borderline inadequate preparation would not be as informative as studies that enrolled subjects with multiple prior poor preparations.

Two previous studies [5, 6] have examined "intensive" bowel preparations in those with previous inadequate preparations. Both used a low fiber diet beginning 3 days-prior to the colonoscopy, a clear liquid diet the day-prior and bisacodyl 10 mg the evening-prior. One prospective study [5] of 51 patients used 3 L PEG-ELS given as a split-dose and found 10% of patients had an inadequate preparation on the subsequent colonoscopy. Another prospective, randomized trial [6] of 239 patients compared 2 preparations given as split-doses with 272 g of PEG in 4 L of water (81% adequately cleansed) superior (*p* = 0.01) to 2 L PEG and ascorbic acid (67% adequately cleansed). Both studies had significant methodologic limitations including: (1) the efficacy of the baseline inadequate preparation was not documented; (2) the “intensive” preparations were no different than standard-dose regimens in use today; (3) 10% to 33% of subjects had inadequate preparations with many of the "adequately" cleansed colons containing residual fecal material; (4) the failed preparations were given as a day-prior dose which is felt by many to be inferior to split dosing [1, 3]; and (5) the number of times each subject had inadequate preparations was not documented.

In our study, 18 of 79 (22.8%) previous inadequate preparations (excluding the unknown ones) were given as a split-dose which might suggest that they were not highly efficacious. 85.5% of subjects had their last inadequate preparations in our clinic with all these colonoscopies performed since 2008. Since 2008, we have enrolled our patients in a series of 3 bowel preparation studies that documented the efficacy of the preparations we used during those eras. Before 2008, 4 L sulfate-free PEG-ELS given the day-prior was our standard preparation with a demonstrated efficacy of 94.5% [11] in our clinic. From 2008 to 2013, 306 g of PEG and Gatorade given the day-prior was the standard preparation with demonstrated efficacy of 97.0% [8, 11]. Since 2014, either 306 g of PEG and Gatorade given as a split-dose or 357 g of PEG and Gatorade given the day-prior have been our standard preparations with demonstrated efficacy of 98.5% [8]. It is less certain how efficacious the preparations were of the 14.5% of subjects who had their most recent inadequately colonoscopy preparations elsewhere.

Our subjects were a very select group with very difficult to cleanse colons because: (1) most had already failed highly efficacious preparations; (2) over the past 12 years, patients with previous preparations that were borderline inadequate received 374 g of PEG and Gatorade given as a split-dose which seemed to work well and those who were successful with this preparation were ineligible to enroll in this study; and (3) 27.1% of subjects had 2 to 4 previous inadequate preparations.

Subjects were significantly older than controls which was expected since they required a previous inadequate colonoscopy preparation to qualify for the study. Compared to controls, subjects were less likely to be undergoing a screening examination and more likely to have polyps found which suggests that a higher risk, more motivated group of patients chose to have a subsequent colonoscopy after an inadequate preparation.

This study did not address the safety and efficacy of a Step 3 bowel preparation (2.5 times the standard-dose of PEG) since no subjects requiring this very high-dose preparation were seen during the recruitment period. Before this study began, we used protocols similar to Step 3 for a few patients who received PEG-ELS and MagCitrate. To date, we have not encountered a patient who had an inadequate Step 2 preparation (2.0 times the standard-dose of PEG). All our subjects used PEG and Gatorade as their bowel cleansing solutions; therefore, this study does not address the safety and efficacy of high doses of other bowel cleansing solutions.

There were some limitations to this study. (1) The study was not a randomized trial and subjects might be more careful to follow any cleansing protocol after a previous inadequate preparation. (2) Only 7 patients with renal insufficiency were enrolled. (3) The study was not blinded. (4) Historical controls were used.

## Conclusions

Step 1 (1.5 times the standard-dose of PEG) and Step 2 (2.0 times the standard-dose of PEG) preparations from our multi-step escalating high-dose extended cleansing protocol using PEG and Gatorade were highly efficacious, safe, well tolerated and well accepted by subjects whose previous colon preparations were inadequate.

This is the first prospective study looking at high-dose bowel preparation solutions for patients who previously had an inadequate bowel preparations for colonoscopy. Bowel preparation solutions have been sold in fixed-dose packages with a "one-size-fit-all" dosing approach for the past 40 years. With this study, we continue to show how the flexible dosing of PEG and Gatorade allows the needs of a diverse patient population to be met. Patients willing to consume a split-dose preparation do well with 306 g of PEG, patients who want a day-prior preparation require 357 g of PEG and we have observed patients with a history of a borderline inadequate preparation doing well with 374 g of PEG given as a spit-dose. Now we report 459 g of PEG given as an extended split-dose preparation works well for those who failed standard dose preparations and 612 g of PEG works well for those who failed 1.5 times the standard preparation.

## Supplementary Information

**Additional file 1**. Doctor Questionnaire.

**Additional file 2**. Instructions for Bowel Preparation for Colonoscopy.

**Additional file 3**. Patient Questionnaire.

**Additional file 4**. High Dose Preparation Solutions for Difficult to Clean Colons.

## Data Availability

All data generated or analyzed during this study are included in this published article as an EXCEL file in Additional file 4.

## Abbreviations

BMP: Basic metabolic panel
BPS: Bowel preparation scale
ELS: Electrolyte solution
PEG: Polyethylene glycol 3350
SF: Sulfate free
