# Supplementary material for: High-dose polyethylene glycol-3350 and gatorade solutions for patients with previous inadequate bowel preparations for colonoscopy are safe and effective
Source: BMC Gastroenterol. 2023 May 11;23:146. doi: 10.1186/s12876-023-02663-0 (PMC10173556; doi:10.1186/s12876-023-02663-0)
Supplement: Supplementary file 1 — Additional file 1. Doctor Questionnaire. [file 12876_2023_2663_MOESM1_ESM.docx]

**Safety and Efficacy of High Dose Bowel Preparation Solutions for Patients with Difficult To Clean Colons for Colonoscopy**

**Doctor Questionnaire**

**Patient Name: __________________________________ Patient Study ID #: _______________**

**Age: ____________ Sex:** M or F **Race**: C AA H Other **MD:** G R

**Clinical Site:** HH GS LaG GS-Office **Scheduled Starting Time:** ______________

**Indication:** Screening h/o Polyps h/o Colon Cancer FH Colon Neoplasia Rectal Bleeding

Long Standing IBD Anemia Other: ____________________________________

**Findings:** ______________________________________________________________________________

**Previous Colon Resection?** Yes or No **If Yes, Site of Resection: ________________________**

**Was the Cecum Reached?** Yes or No **If No, Extent of Colonoscopy: ___________________**

**Boston Bowel Preparation Scale (BBPS)**

0=Unprepared colon segment with stool that cannot be cleared

1=Portion of mucosa in segment seen **after** cleaning, but other areas not seen due to retained material

2=Minor residual material **after** cleaning, but mucosa of segment generally well seen

3=Entire mucosa of segment well seen **after** cleaning

BBPS **Right Colon**: 0 1 2 3

BBPS **TV Colon**: 0 1 2 3 (BBPS 2 --- corresponds to CBPS 10)

BBPS **Left Colon**: 0 1 2 3 (BBPS 3 --- corresponds to CBPS 11 or 12)

**Chicago Bowel Preparation Scale (CBPS)**

0=Unprepared colon segment with stool that cannot be cleared (15+% of the mucosa not seen)

5=Portion of mucosa in segment seen **after** cleaning, but up to 15% of the mucosa not seen due to retained material

10=Minor residual material **after** cleaning, but mucosa of segment generally well seen

11=Entire mucosa of segment well seen **after** cleaning

12=Entire mucosa of segment well seen **without** washing (suctioning of liquid allowed)

CBPS **Right Colon**: 0 5 10 11 12

CBPS **TV Colon**: 0 5 10 11 12

CBPS **Left Colon**: 0 5 10 11 12

CBPS **Fluid Whole Colon:** 3=Lots of fluid (300+ cc) 2=Moderate fluid (151-300 cc)

1=Minimal fluid (51-150 cc) 0=Little fluid (less than 50 cc)

**Volume of liquid was removed from the colon (cc)? _____________ Wash used (cc)? _____________**

**Did you consider the preparation adequate for a good quality colonoscopy (able to see at least 95% of the mucosa)?** Yes or No

**Would you recommend the same preparation or more preparation for the next colonoscopy?** Same or More

**Previous Preparations and Results** _________________________________________________________
