# Supplementary material for: High-dose polyethylene glycol-3350 and gatorade solutions for patients with previous inadequate bowel preparations for colonoscopy are safe and effective
Source: BMC Gastroenterol. 2023 May 11;23:146. doi: 10.1186/s12876-023-02663-0 (PMC10173556; doi:10.1186/s12876-023-02663-0)
Supplement: Supplementary file 2 — Additional file 2. Instructions for Bowel Preparation for Colonoscopy. [file 12876_2023_2663_MOESM2_ESM.docx]

Instructions for Bowel Preparation for Colonoscopy

306 g MiraLAX Split-Dose (standard dose)

Three Days Before Colonoscopy

Omit fruits and vegetables with seeds or foods that are high in fiber from your diet. Examples include: cucumbers, tomatoes, corn, raspberries, whole grain breads and cereals, popcorn, and nuts.

**Do not** take Metamucil or similar fiber products. Do not take iron tablets, aspirin, or Plavix for 3 days before the procedure. NSAID medications such as ibuprofen (Advil, Motrin) and naproxen (Aleve, Naprosyn) should not be taken for 24 hours prior to your procedure. If you are on Coumadin, your doctor usually will direct you to hold the medication for 3 days. If you have a history of coronary artery disease (heart attack, heart stent, etc.) or stroke, you may continue to take a baby aspirin daily.

Have your prescription for Polyethylene Glycol (generic MiraLAX) filled if your insurance covers it. Otherwise, purchase a 510 gram bottle of MiraLAX which is available over-the-counter. Also purchase 64 oz of Gatorade and refrigerate it. Any flavor (including G2 which is low sugar Gatorade) is fine as long as it is not red in color.

**One Day Before Colonoscopy**

Drink **clear liquids** **only** all day. Examples include: water, black coffee, tea, soda, apple juice, beef broth, chicken or vegetable broth, grape juice, Gatorade, Jell-O or Popsicles. Please exclude liquids that are **RED** in color. Diabetics may drink Glucerna (no fiber) only if their sugars run low. Drink PLENTY of fluids throughout the day to prevent dehydration. You will feel better and will make it easier for your nurse to find a vein for your I.V. in the morning!

At 6:00 PM, mix 9 capfuls (153 grams) of MiraLAX into 32 oz of Gatorade. Start drinking the liquid at the rate of one 8 oz glass every ten to twenty minutes. Drinking at this rate will give you the best cleaning results. If you find the taste unpleasant, you might want to rinse your mouth out with water or mouthwash between glasses. Sometimes drinking the liquid through a straw helps.

The next morning, 4 hours before your colonoscopy is scheduled, mix an additional 9 capfuls (153 grams) of MiraLAX into 32 oz of Gatorade. Start drinking the liquid at the rate of one 8 oz glass every ten to twenty minutes.

You must drink the entire 32oz both the evening before and the morning of your colonoscopy (64 oz total)! Drinking any large volume of cold fluids may cause chills. To reduce this risk, pour several 8 oz glasses at once and allow them to warm slightly before drinking them.

You may feel very full or bloated until your bowels begin to move. The first bowel movement usually occurs in about an hour, but this can vary. If you develop significant nausea or vomiting, take a break from drinking for about 30 minutes and then try again when you feel better. If you still cannot tolerate the drink, call our office (630-969-1167) and have the doctor on-call paged. If you don’t hear from him within 30 minutes, have him paged again.

After you finish drinking the preparation, you may have clear liquids up until 2 hours prior to your examination. Remember that you’ll feel better with extra fluids and your I.V. will be easier to start. You will continue to have bowel movements for some time. Your final stools should be very watery, yellow in color, maybe a little cloudy but you should be able to see the bottom of the toilet. If your stools are **not clear**, you should give yourself 2 to 3 adult-sized Fleet enemas which can be purchased without a prescription at any drug store.

**Please bring a list of current medications with you**. Ladies! Please come with one fingernail on your right hand free of nail polish so we can monitor your oxygen level.

Instructions for Bowel Preparation for Colonoscopy

459 g MiraLAX Split-Dose (step 1)

Three Days Before Colonoscopy

Omit fruits and vegetables with seeds or foods that are high in fiber from your diet. Examples include: cucumbers, tomatoes, corn, raspberries, whole grain breads and cereals, popcorn, and nuts.

**Do not** take Metamucil or similar fiber products. Do not take iron tablets, aspirin, or Plavix for 3 days before the procedure. NSAID medications such as ibuprofen (Advil, Motrin) and naproxen (Aleve, Naprosyn) should not be taken for 24 hours prior to your procedure. If you are on Coumadin, your doctor usually will direct you to hold the medication for 3 days. If you have a history of coronary artery disease (heart attack, heart stent, etc.) or stroke, you may continue to take a baby aspirin daily.

Have your prescription for Polyethylene Glycol (generic MiraLAX) filled if your insurance covers it. Otherwise, purchase a 510 gram bottle of MiraLAX which is available over-the-counter. Also purchase three 32 oz bottles of Gatorade (96 oz total) and refrigerate it. Any flavor (including G2 which is low sugar Gatorade) is fine as long as it is not red in color.

**One Day Before Colonoscopy**

Drink **clear liquids** **only** all day. Examples include: water, black coffee, tea, soda, apple juice, beef broth, chicken or vegetable broth, grape juice, Gatorade, Jell-O or Popsicles. Please exclude liquids that are **RED** in color. Diabetics may drink Glucerna (no fiber) only if their sugars run low. Drink PLENTY of fluids throughout the day to prevent dehydration. You will feel better and will make it easier for your nurse to find a vein for your I.V. in the morning!

At noon, mix 3 capfuls (51grams) of the MiraLAX into 12 oz of any clear liquid and drink the liquid. Continue with lots of clear liquids until 2:00 PM, and then fast for 3 hours. You may or may not have a bowel movement during the afternoon.

At 5:00 PM, mix 15 capfuls (255 grams or 1 ¼ cups) of MiraLAX into 64 oz of Gatorade. Start drinking the liquid at the rate of one 8 oz glass every ten to twenty minutes. Drinking at this rate will give you the best cleaning results. If you find the taste unpleasant, you might want to rinse your mouth out with water or mouthwash between glasses. Sometimes drinking the liquid through a straw helps.

The next morning, 4 hours before your colonoscopy is scheduled, mix an additional 9 capfuls (153 grams) of MiraLAX into 32 oz of Gatorade. Start drinking the liquid at the rate of one 8 oz glass every ten to twenty minutes.

You must drink the entire 32oz both the evening before and the morning of your colonoscopy (64 oz total)! Drinking any large volume of cold fluids may cause chills. To reduce this risk, pour several 8 oz glasses at once and allow them to warm slightly before drinking them.

You may feel very full or bloated until your bowels begin to move. The first bowel movement usually occurs in about an hour, but this can vary. If you develop significant nausea or vomiting, take a break from drinking for about 30 minutes and then try again when you feel better. If you still cannot tolerate the drink, call our office (630-969-1167) and have the doctor on-call paged. If you don’t hear from him within 30 minutes, have him paged again.

After you finish drinking the preparation, you may have clear liquids up until 2 hours prior to your examination. Remember that you’ll feel better with extra fluids and your I.V. will be easier to start. You will continue to have bowel movements for some time. Your final stools should be very watery, yellow in color, maybe a little cloudy but you should be able to see the bottom of the toilet. If your stools are **not clear**, you should give yourself 2 to 3 adult-sized Fleet enemas which can be purchased without a prescription at any drug store.

**Please bring a list of current medications with you**. Ladies! Please come with one fingernail on your right hand free of nail polish so we can monitor your oxygen level.

Instructions for Bowel Preparation for Colonoscopy

612 g MiraLAX Split-Dose (step 2)

Three Days Before Colonoscopy

Omit fruits and vegetables with seeds or foods that are high in fiber from your diet. Examples include: cucumbers, tomatoes, corn, raspberries, whole grain breads and cereals, popcorn, and nuts.

**Do not** take Metamucil or similar fiber products. Do not take iron tablets, aspirin, or Plavix for 3 days before the procedure. NSAID medications such as ibuprofen (Advil, Motrin) and naproxen (Aleve, Naprosyn) should not be taken for 24 hours prior to your procedure. If you are on Coumadin, your doctor usually will direct you to hold the medication for 3 days. If you have a history of coronary artery disease (heart attack, heart stent, etc.) or stroke, you may continue to take a baby aspirin daily.

Have your prescription for Polyethylene Glycol (generic MiraLAX) filled if your insurance covers it. Otherwise, purchase two 510 gram bottles of MiraLAX which is available over-the-counter. Also purchase four 32 oz bottles of Gatorade (128 oz total) and refrigerate it. Any flavor (including G2 which is low sugar Gatorade) is fine as long as it is not red in color.

**One Day Before Colonoscopy**

Drink **clear liquids** **only** all day. Examples include: water, black coffee, tea, soda, apple juice, beef broth, chicken or vegetable broth, grape juice, Gatorade, Jell-O or Popsicles. Please exclude liquids that are **RED** in color. Diabetics may drink Glucerna (no fiber) only if their sugars run low. Drink PLENTY of fluids throughout the day to prevent dehydration. You will feel better and will make it easier for your nurse to find a vein for your I.V. in the morning!

At noon, mix 9 capfuls (153 grams) of the MiraLAX into 32 oz of Gatorade. Continue with lots of clear liquids until 2:00 PM, and then fast for 3 hours. You may or may not have a bowel movement during the afternoon.

At 5:00 PM, mix 18 capfuls (306 grams) of MiraLAX into 64 oz of Gatorade. Start drinking the liquid at the rate of one 8 oz glass every ten to twenty minutes. Drinking at this rate will give you the best cleaning results. If you find the taste unpleasant, you might want to rinse your mouth out with water or mouthwash between glasses. Sometimes drinking the liquid through a straw helps.

The next morning, 4 hours before your colonoscopy is scheduled, mix an additional 9 capfuls (153 grams) of MiraLAX into 32 oz of Gatorade. Start drinking the liquid at the rate of one 8 oz glass every ten to twenty minutes.

You must drink the entire 32oz both the evening before and the morning of your colonoscopy (64 oz total)! Drinking any large volume of cold fluids may cause chills. To reduce this risk, pour several 8 oz glasses at once and allow them to warm slightly before drinking them.

You may feel very full or bloated until your bowels begin to move. The first bowel movement usually occurs in about an hour, but this can vary. If you develop significant nausea or vomiting, take a break from drinking for about 30 minutes and then try again when you feel better. If you still cannot tolerate the drink, call our office (630-969-1167) and have the doctor on-call paged. If you don’t hear from him within 30 minutes, have him paged again.

After you finish drinking the preparation, you may have clear liquids up until 2 hours prior to your examination. Remember that you’ll feel better with extra fluids and your I.V. will be easier to start. You will continue to have bowel movements for some time. Your final stools should be very watery, yellow in color, maybe a little cloudy but you should be able to see the bottom of the toilet. If your stools are **not clear**, you should give yourself 2 to 3 adult-sized Fleet enemas which can be purchased without a prescription at any drug store.

**Please bring a list of current medications with you**. Ladies! Please come with one fingernail on your right hand free of nail polish so we can monitor your oxygen level.
