# Supplementary material for: High-dose polyethylene glycol-3350 and gatorade solutions for patients with previous inadequate bowel preparations for colonoscopy are safe and effective
Source: BMC Gastroenterol. 2023 May 11;23:146. doi: 10.1186/s12876-023-02663-0 (PMC10173556; doi:10.1186/s12876-023-02663-0)
Supplement: Supplementary file 3 — Additional file 3. Patient Questionnaire. [file 12876_2023_2663_MOESM3_ESM.docx]

**Safety and Efficacy of High Dose Bowel Preparation Solutions for Patients with Difficult To Clean Colons for Colonoscopy**

**Patient Questionnaire**

**Patient Name: __________________________________ Patient Study ID #: _______________**

**While cleaning yourself out for the colonoscopy, did any of the following problems occur:**

**Nausea:** 0=none 1=mild 2=moderate 3=severe

**Vomiting:** 0=none 1=mild 2=moderate 3=severe

**Abdominal Cramps/Pain:** 0=none 1=mild 2=moderate 3=severe

**Bloating:** 0=none 1=mild 2=moderate 3=severe

**Did any other problems occur during your preparation for your colonoscopy?**

**How much of your preparation were you able to drink?** All or _______________________________

Or

**How many ounces of liquid were left after you were done drinking**? None or ______________________

**Did you need to call the doctor for advice or help?** Yes or No

**Did you need to take any enemas in the morning of your colonoscopy?** Yes or No **How Many?** ______

**Did you need to change your medicine to clean out your colon because you could not drink it all?** Yes or No

**Did you wake up in the middle of the night to have a bowel movement?** Yes or No

**If you needed to have another colonoscopy in the future would you want to use the same liquid preparation?**

Yes or No

**How difficult was it to drink the liquid?** 0=Easy 1=Mildly Difficult 2=Moderately Difficult 3=Very Difficult

**Overall, how difficult was the preparation?** 0=Easy 1=Mildly Difficult 2=Moderately Difficult 3=Very Difficult

**With your last colonoscopy:**

1. **What did you use to clean yourself out last time?** _________________________________

If you don’t remember the name, describe what it was. For example, “gallon of liquid”, “pills”, etc.

1. **How did your current preparation compare to the previous one? The prep I took this time was:** Easier About The Same More Difficult **compared to the previous one.**

**Do you take medications for constipation?** Yes or No

**If yes, what medications do you take and how often? ________**________________________________
